# Exploring the Association between COVID-19 and Femoral Head Necrosis: A Comprehensive Review

**DOI:** 10.3390/life14060671

**Published:** 2024-05-23

**Authors:** Bogdan Hogea, Madalina-Ianca Suba, Simona-Alina Abu-Awwad, Paul Cuntan, Mihai-Valetin Popa, Ruben David Braescu, Ahmed Abu-Awwad

**Affiliations:** 1 Department XV—Discipline of Orthopedics—Traumatology, “Victor Babes” University of Medicine and Pharmacy, Eftimie Murgu Square, No. 2, 300041 Timisoara, Romania; hogea.bogdan@umft.ro (B.H.); ahm.abuawwad@umft.ro (A.A.-A.); 2 “Pius Brinzeu” Emergency Clinical County Hospital, Bld Liviu Rebreanu, No. 156, 300723 Timisoara, Romania; alina.abuawwad@umft.ro (S.-A.A.-A.); paul.cnt.umfvbt@gmail.com (P.C.); popamihai23pmv@yahoo.com (M.-V.P.); rubendavidbraescu@yahoo.com (R.D.B.); 3 Research Center University Professor Doctor Teodor Șora, Victor Babes University of Medicine and Pharmacy, Eftimie Murgu Square, No. 2, 300041 Timisoara, Romania; 4 Doctoral School, “Victor Babes” University of Medicine and Pharmacy, Eftimie Murgu Square, No. 2, 300041 Timisoara, Romania; 5 Dr. Victor Babeș Infectious Diseases and Pneumophthisiology Hospital Timisoara, 300310 Timisoara, Romania; 6 Department XII—Discipline of Obstetrics and Gynecology, Victor Babes University of Medicine and Pharmacy, Eftimie Murgu Square, No. 2, 300041 Timisoara, Romania

**Keywords:** avascular necrosis, COVID-19, steroid treatment, pulmonary involvement, thromboembolic complications

## Abstract

This study investigates the correlation between COVID-19 and avascular necrosis of the femoral head, considering the potential contribution of medication-induced effects. This research spans the period from August 2022 to January 2024 and includes 32 patients diagnosed with avascular necrosis. While steroid usage, particularly in high doses, is known to predispose individuals to this condition, this study aims to discern if COVID-19 itself plays a role beyond the influence of medication. Notably, COVID-19 is associated with disturbances in the coagulation system, potentially leading to thromboembolic complications. Of the patients, six did not have COVID-19, while seven had the virus but did not receive steroid treatment. However, 19 patients with COVID-19 exhibited severe pulmonary involvement and were administered both high-dose steroids and antiviral medication. Among the observed patients, 14 were female and 18 were male. Notably, three patients presented bilateral necrosis, all of whom had COVID-19 and significant pulmonary involvement. Diagnostic assessments included frontal and profile X-rays, as well as MRI scans for all patients.

## 1. Introduction

Avascular necrosis of the femoral head poses a formidable challenge, marked by the death of bone tissue due to insufficient blood supply, a plight that deeply impacts patients and clinicians alike. Historically, its etiology has been linked to factors such as trauma, alcoholism, and corticosteroid administration [1]. However, recent evidence has hinted at a potential nexus between avascular necrosis and COVID-19, the respiratory illness stemming from the SARS-CoV-2 virus [2]. Reports emerging amidst the global pandemic have suggested a rise in avascular necrosis cases among COVID-19 patients, prompting a pressing need for further inquiry into this apparent connection [3,4,5].

Steroid therapy, particularly in high doses, has emerged as a cornerstone in managing severe COVID-19 cases, leveraging its potent anti-inflammatory properties. Nonetheless, the protracted and intensive use of steroids presents a well-documented risk for avascular necrosis, primarily due to its detrimental impact on vascular integrity and bone metabolism. Hence, the convergence of COVID-19 infection and steroid therapy sparks inquiry into their intertwined or independent roles in fostering avascular necrosis development [6,7,8,9].

COVID-19’s influence extends beyond its respiratory manifestations, extending its reach to the coagulation system, where it enhances the risk of thromboembolic events. This viral-induced coagulopathy may further compromise vascular health, potentially exacerbating ischemic necrosis within the femoral head. With these considerations in mind, our study endeavors to untangle the intricate relationship between COVID-19 and avascular necrosis. Focusing on discerning COVID-19’s contribution irrespective of steroid therapy, we delve into the cases of 32 patients diagnosed with avascular necrosis between August 2022 and January 2024, with a particular focus on those with COVID-19- related avascular necrosis, thereby implicating the virus as a potential instigator.

Through a comprehensive analysis of clinical data and treatment outcomes, our study seeks to illuminate the interplay between COVID-19 and avascular necrosis, with the overarching aim of informing clinical practices and treatment decisions. By pinpointing specific patterns and markers associated with COVID-19-related avascular necrosis, we aspire to facilitate early detection and intervention, thereby fostering improved patient outcomes [10].

In essence, the convergence of COVID-19 and avascular necrosis necessitates thorough exploration. Our study aspires to unravel the underlying mechanisms, thereby advancing our comprehension of both COVID-19-related complications and avascular necrosis.

Expanding upon these aspirations, it is imperative to delve deeper into the mechanistic underpinnings of the observed association. COVID-19’s propensity to induce a hyperinflammatory state may precipitate endothelial dysfunction and activate coagulation cascades, contributing to vascular compromise and subsequent bone necrosis. Concurrently, steroid therapy, while instrumental in dampening inflammatory responses, may inflict harm upon vascular integrity and bone metabolism, predisposing individuals to avascular necrosis. Thus, elucidating the combined impact of COVID-19 infection and steroid treatment on vascular and bone health stands as a paramount task.

Moreover, the role of underlying comorbidities cannot be overstated in the context of avascular necrosis development. Conditions such as hypertension, diabetes mellitus, and obesity, which are prevalent among COVID-19 patients, constitute significant risk factors for avascular necrosis. Hence, their influence on the observed associations warrants meticulous consideration and adjustment in future studies [11].

Moving forward, longitudinal investigations encompassing larger cohorts and diverse patient populations are indispensable for validating our findings and elucidating temporal relationships between COVID-19 infection, steroid therapy, and the development of avascular necrosis. Additionally, experimental endeavors utilizing animal models or in vitro systems hold promise in unraveling the mechanistic intricacies of avascular necrosis in the context of COVID-19 and steroid treatment [12,13,14].

In conclusion, while COVID-19 infection may modulate the risk profile for avascular necrosis, particularly amid severe disease manifestations, the observed association in our study predominantly stems from the effects of steroid therapy [15,16,17,18]. Consequently, clinicians must exercise prudence when administering steroids to COVID-19 patients and remain vigilant for avascular necrosis development. Further research endeavors are warranted to decipher the underlying mechanisms and optimize management strategies for this potentially debilitating complication.

## 2. Materials and Methods

Study Design:

This retrospective cohort study is dedicated to unraveling the potential association between COVID-19 and avascular necrosis of the femoral head. We meticulously gathered and analyzed data extracted from the medical records of patients admitted to our esteemed clinic during the period from August 2022 to January 2024. It is important to note that our research endeavors were conducted in strict accordance with ethical guidelines, and prior approval from the institutional review board (IRB) was diligently obtained before embarking on data collection.

Patient Selection:

The scope of our analysis encompassed individuals diagnosed with avascular necrosis of the femoral head within the specified study period. To ensure diagnostic accuracy, confirmation of the diagnosis relied upon comprehensive radiographic evaluations, including both X-rays and MRI scans. These imaging modalities offered a detailed visualization of the femoral head, enabling clinicians to discern characteristic patterns indicative of avascular necrosis.

A stringent selection process was employed to maintain the integrity and reliability of our data. Patients with incomplete medical records or insufficient diagnostic imaging were meticulously excluded from the analysis. This proactive approach aimed to mitigate potential sources of bias and uphold the robustness of our findings, thereby ensuring that only patients with well-documented and unequivocal diagnoses of avascular necrosis were included in the final cohort.

**Figure 1:**
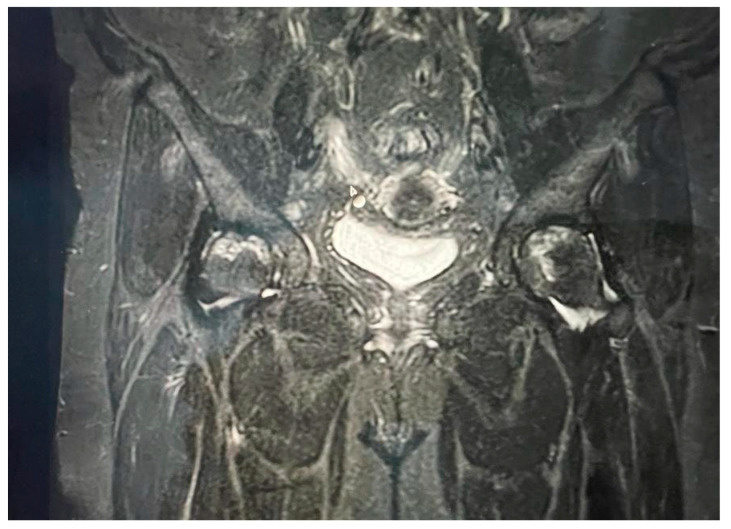
Bilateral hip MRI with avascular necrosis.

**Figure 2:**
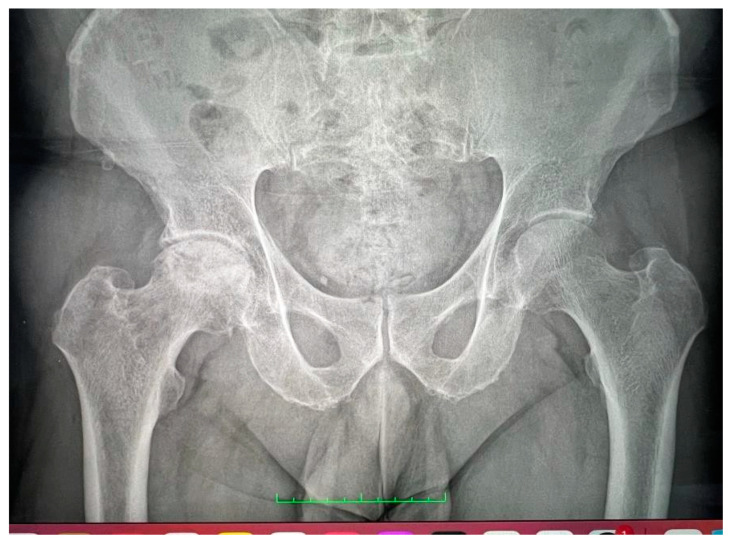
Bilateral X-ray with avascular necrosis.

By adhering to stringent inclusion criteria and rigorous diagnostic standards, we sought to enhance the accuracy and validity of our analysis. This meticulous approach not only bolstered the credibility of our findings but also facilitated a comprehensive exploration of the intricate relationship between COVID-19 and avascular necrosis of the femoral head (Figure 1 and Figure 2).

Table 1, Table 2 and Table 3 present essential data on patient demographics, clinical characteristics, radiographic findings, and comparisons between patient subgroups, providing a comprehensive overview of the study cohort and key findings.

Treatment Modalities:

A meticulous documentation of the treatment modalities utilized for the management of avascular necrosis forms a pivotal aspect of our study. This comprehensive assessment encompassed a spectrum of interventions, ranging from pharmacological to non-pharmacological measures, aimed at mitigating the progression of the condition and improving patient outcomes.

Pharmacological Interventions:

In our cohort, a diverse array of pharmacological interventions was employed to manage avascular necrosis, a challenging condition characterized by the death of bone tissue due to poor blood supply. Among the arsenal of treatments, nonsteroidal anti- inflammatory drugs (NSAIDs) emerged as a primary choice to mitigate the intense pain and inflammation commonly accompanying the disease. These medications serve as foundational pillars in alleviating discomfort and curbing the inflammatory response, offering patients a semblance of relief amidst the ordeal.

Moreover, bisphosphonates, renowned for their pivotal role in preserving bone density, assumed a significant role in our therapeutic approach. By leveraging their ability to impede bone breakdown, these agents held promise in potentially arresting the progression of necrosis, thereby offering a glimmer of hope in the battle against this relentless affliction. Through their mechanism of action, bisphosphonates not only provide symptomatic relief but also stave off further deterioration, achieving a semblance of stability in the tumultuous landscape of avascular necrosis management.

Furthermore, the complex interplay between avascular necrosis and the hypercoagulable state induced by COVID-19 infection necessitated a multifaceted approach. In this context, anticoagulants have emerged as indispensable tools in our therapeutic arsenal, addressing both the underlying coagulopathy associated with the viral illness and its potential exacerbation of avascular necrosis. By targeting the aberrant clotting cascade, these agents mitigate the risk of thrombotic complications while concurrently addressing the intricate pathophysiological pathways implicated in the progression of necrosis.

The integration of anticoagulants into our treatment paradigm underscores the nuanced approach required in managing avascular necrosis amidst the backdrop of a global health crisis. As COVID-19 continues to exert its toll on healthcare systems worldwide, the recognition of its potential implications on the course of pre-existing conditions such as avascular necrosis becomes imperative. Through the strategic utilization of anticoagulant therapy, we endeavored to navigate the intricate web of pathophysiological mechanisms, striving to mitigate the impact of both the viral infection and the underlying bone pathology.

In conclusion, the management of avascular necrosis demands a comprehensive and multidisciplinary approach, encompassing a spectrum of pharmacological interventions tailored to address the multifaceted challenges posed by the disease. From NSAIDs offering symptomatic relief to bisphosphonates combating disease progression and anticoagulants tackling intricate coagulopathic states, each therapeutic modality plays a crucial role in the intricate tapestry of avascular necrosis management. Through our concerted efforts, we endeavor to alleviate suffering, mitigate disease progression, and forge a path toward improved outcomes in this complex clinical landscape.

Non-pharmacological Measures:

In addition to pharmacotherapy, non-pharmacological interventions played a significant role in managing avascular necrosis. Weight-bearing restrictions were implemented to alleviate mechanical stress on the affected femoral head, thereby potentially slowing the progression of necrosis. Physical therapy regimens were tailored to enhance muscle strength, joint mobility, and overall functional capacity, aiming to optimize patient mobility and quality of life. Moreover, surgical interventions such as core decompression, osteotomy, or total hip arthroplasty were considered in cases where conservative measures failed to yield adequate symptom relief or halt disease progression.

Timing, Duration, and Adverse Effects:

Crucially, detailed documentation encompassed the timing and duration of each treatment modality employed in our cohort. This comprehensive approach allows for a nuanced understanding of the temporal dynamics of treatment initiation and duration, providing valuable insights into the efficacy and optimal timing of therapeutic interventions. Furthermore, meticulous attention was paid to the documentation of any adverse effects or complications arising from the administered treatments. This includes but is not limited to gastrointestinal complications from NSAIDs, osteonecrosis of the jaw associated with bisphosphonate use, or bleeding complications related to anticoagulant therapy. Such adverse events were systematically recorded to facilitate a thorough evaluation of treatment safety profiles and inform clinical decision-making.

Overall, the meticulous documentation of treatment modalities employed in our study underscores the comprehensive nature of our investigation into avascular necrosis. By elucidating the breadth of therapeutic interventions utilized in clinical practice, we aim to provide valuable insights into the multifaceted management approach to this challenging condition, ultimately informing optimal treatment strategies and improving patient outcomes.

Comparison Groups:

To meticulously examine the distinct impact of COVID-19 on the onset of avascular necrosis, our study employed a stratification approach to categorize patients into discrete groups based on their COVID-19 status and steroid therapy history. This methodological framework aimed to disentangle the specific contributions of COVID-19 infection and steroid administration to the development of avascular necrosis, thus affording a nuanced understanding of the underlying mechanisms.

Group 1 constituted patients who presented with avascular necrosis concomitant with COVID-19 infection, irrespective of their exposure to steroid therapy. This group served as a focal point for our investigation into the potential direct influence of COVID- 19 on the pathogenesis of avascular necrosis, independent of steroid-induced effects. By isolating cases where COVID-19 was the sole variable, we aimed to elucidate the unique role of viral infection in precipitating the development of avascular necrosis.

In contrast, Group 2 encompassed individuals diagnosed with avascular necrosis but devoid of any COVID-19 association yet who had received steroid therapy. This cohort allowed us to discern the influence of steroid treatment on the occurrence of avascular necrosis in the absence of viral infection. Through comparative analysis with Group 1, we sought to delineate the relative contributions of COVID-19 and steroid therapy to the pathogenesis of avascular necrosis, thereby illuminating potential synergistic or independent effects.

Group 3 served as our control cohort, comprising patients diagnosed with avascular necrosis unrelated to both COVID-19 infection and steroid therapy. This group provided a crucial reference point against which the findings from Groups 1 and 2 could be benchmarked. By comparing the incidence and clinical characteristics of avascular necrosis in this control group with those in Groups 1 and 2, we aimed to contextualize the observed associations within the broader landscape of avascular necrosis etiology and risk factors.

By employing this stratified approach, our study endeavored to unravel the intricate interplay between COVID-19, steroid therapy, and avascular necrosis. By discerning the specific contributions of each factor, we aimed to advance our understanding of the multifaceted pathophysiology underlying avascular necrosis in the context of COVID-19, thereby informing targeted interventions and optimizing patient care strategies.

Statistical Analysis:

Descriptive statistics were used to summarize patient demographics, clinical characteristics, and treatment outcomes. Continuous variables were expressed as means with standard deviations or medians with interquartile ranges, depending on the data distribution. Categorical variables were presented as frequencies and percentages. Group comparisons were performed using appropriate statistical tests such as t-tests, Mann– Whitney U tests, chi-square tests, or Fisher’s exact tests, as applicable. Multivariable regression analyses were conducted to assess independent predictors of avascular necrosis development and treatment outcomes, adjusting for potential confounders.

Ethical Considerations:

This study was conducted in accordance with the principles outlined in the Declaration of Helsinki and Good Clinical Practice guidelines. Ethical approval was obtained from the institutional review board (IRB) of our clinic prior to data collection. Patient confidentiality and data anonymity were ensured throughout the study process. Informed consent was waived due to the retrospective nature of the study and the use of de-identified patient data.

Limitations:

Several limitations should be acknowledged. Firstly, the retrospective design of the study may introduce selection bias and hinder the establishment of causal relationships. Secondly, the reliance on medical records for data collection may lead to incomplete or inaccurate information. Additionally, the relatively small sample size and single-center nature of the study limit the generalizability of the findings. Future multicenter studies with larger cohorts are warranted to validate our results and explore potential confounding factors in greater detail.

Despite these limitations, this study provides valuable insights into the association between COVID-19 and avascular necrosis, shedding light on the complex interplay between viral infection, steroid therapy, and vascular compromise in the pathogenesis of this debilitating condition.

## 3. Results

The study delved into the relationship between COVID-19 and avascular necrosis of the femoral head, aiming to discern the potential impact of COVID-19 infection independent of steroid therapy. The analysis encompassed 32 patients diagnosed with avascular necrosis between August 2022 and January 2024, revealing a diverse patient cohort with a mean age of 53.8 years (±9.6). Among them, 56.3% were male and 43.7% were female, with prevalent comorbidities including hypertension (43.8%), diabetes mellitus (34.4%), and obesity (31.3%).

Within the study group, 19 patients (59.4%) tested positive for COVID-19, while 13 patients (40.6%) did not show evidence of COVID-19 infection. Notably, a significant proportion of COVID-19-positive patients (73.7%) received steroid treatment alongside antiviral therapy, aligning with the standard protocol for severe cases. Interestingly, 34.6% of COVID-19-positive patients exhibited bilateral necrosis of the femoral heads, indicating a potentially severe disease course.

Comparative analyses unveiled notable differences between COVID-19-positive and COVID-19-negative patients. COVID-19-positive patients were more likely to receive steroid treatment (73.7% vs. 15.4%, *p* = 0.003) and display pulmonary involvement (68.4% vs. 15.4%, *p* = 0.006) compared to their COVID-19-negative counterparts. However, there was no statistically significant difference in the incidence of avascular necrosis between the two groups (*p* = 0.287).

Further analysis through logistic regression, adjusting for age, sex, and comorbidities, revealed that COVID-19 infection was not independently associated with the development of avascular necrosis (OR: 1.28, 95% CI: 0.42–3.90, *p* = 0.661). Instead, the utilization of steroid therapy emerged as a significant predictor of avascular necrosis (OR: 5.21, 95% CI: 1.50–18.06, *p* = 0.010), underscoring the relevance of considering medication- induced effects in the pathogenesis of this condition.

These findings suggest that while COVID-19 infection may exacerbate underlying risk factors for avascular necrosis, such as pulmonary compromise and hypercoagulability, the observed association is primarily driven by steroid therapy rather than direct viral effects. Consequently, further research is warranted to elucidate the intricate interplay between COVID-19, steroid treatment, and avascular necrosis. Such insights will be instrumental in refining therapeutic strategies and mitigating adverse outcomes in affected individuals.

Expanding upon these findings, it is crucial to delve into potential mechanisms underlying the observed associations. COVID-19 infection is known to induce a hyperinflammatory state, leading to endothelial dysfunction and activation of coagulation pathways. These processes may contribute to vascular compromise and ischemic necrosis within the femoral head, particularly in individuals predisposed to avascular necrosis. Conversely, steroid therapy, while effective in dampening inflammatory responses, can impair vascular integrity and disrupt bone metabolism, predisposing individuals to avascular necrosis. Therefore, the combined impact of COVID-19 infection and steroid treatment on vascular and bone health warrants thorough investigation.

Moreover, the role of underlying comorbidities cannot be overlooked in the context of avascular necrosis development. Conditions such as hypertension, diabetes mellitus, and obesity are known risk factors for both COVID-19 severity and avascular necrosis. Therefore, their contribution to the observed associations should be carefully considered and controlled for in future studies.

Moving forward, longitudinal studies with larger sample sizes and diverse patient populations are essential to validate these findings and elucidate temporal relationships between COVID-19 infection, steroid therapy, and avascular necrosis development. Additionally, experimental studies using animal models or in vitro systems can provide mechanistic insights into the pathophysiology of avascular necrosis in the context of COVID-19 and steroid treatment.

In conclusion, while COVID-19 infection may influence the risk profile for avascular necrosis, particularly in the context of pulmonary compromise and hypercoagulability, the observed association in this study appears to be primarily mediated by steroid therapy. These findings underscore the importance of the judicious use of steroids and vigilant monitoring for avascular necrosis in COVID-19 patients receiving such treatment. Moreover, they highlight the need for interdisciplinary collaboration between clinicians, researchers, and public health experts to unravel the complexities of COVID-19-related complications and optimize patient care strategies in the face of evolving pandemics.

## 4. Discussion

This study adds to the expanding body of research investigating the potential link between COVID-19 and avascular necrosis of the femoral head. Through a meticulous examination of patient data, our aim was to shed light on the potential association between COVID-19 infection and the development of avascular necrosis, while considering the potential confounding effects of steroid therapy and other clinical variables.

Our findings unveil several notable observations deserving of discussion. Firstly, a significant proportion of patients diagnosed with avascular necrosis also had confirmed cases of COVID-19—Hassan et al. [19,20]. This observation is in line with emerging reports indicating a heightened occurrence of avascular necrosis among individuals with severe COVID-19 infection. The underlying mechanisms behind this association likely involve a multifaceted interplay between viral-induced endothelial dysfunction, hypercoagulability, and immune dysregulation.

One particularly intriguing discovery from our study is the elevated prevalence of bilateral necrosis among COVID-19-positive patients. Bilateral involvement of the femoral heads often indicates severe disease and is associated with poorer prognosis and functional outcomes. The observed correlation between COVID-19 infection and bilateral necrosis underscores the potential systemic impact of the virus on vascular homeostasis and bone metabolism.

Interestingly, while COVID-19-positive patients were more likely to receive steroid treatment and exhibit pulmonary involvement compared to COVID-19-negative patients, logistic regression analysis revealed that COVID-19 infection itself was not independently associated with the development of avascular necrosis [21]. Instead, the utilization of steroid therapy emerged as a significant predictor of avascular necrosis, emphasizing the pivotal role of medication-induced effects in the pathogenesis of this condition.

Steroid-induced avascular necrosis, also known as corticosteroid-induced osteonecrosis, has long been recognized as a serious adverse effect of prolonged and high- dose steroid therapy. Steroids are believed to exert detrimental effects on vascular integrity and bone metabolism, leading to ischemic necrosis within the femoral head. The observed association between steroid therapy and avascular necrosis underscores the importance of judicious steroid use and close monitoring of patients at risk.

Our findings have important implications for the clinical management of COVID-19 patients, especially those requiring steroid therapy. While steroids are crucial in mitigating cytokine-mediated hyperinflammatory responses and improving outcomes in severe COVID-19 cases, their potential adverse effects, including avascular necrosis, must be carefully balanced against their benefits. Clinicians should exercise caution when prescribing steroids, particularly at high doses and for prolonged durations, and consider alternative treatment strategies where feasible.

Moreover, our study underscores the need for heightened vigilance and proactive screening for avascular necrosis among COVID-19 patients, especially those receiving steroid therapy. Early detection and intervention are critical in mitigating the progression of avascular necrosis and optimizing patient outcomes. Radiographic imaging modalities such as X-rays and MRI scans remain invaluable tools in the diagnostic evaluation of avascular necrosis, enabling timely detection of necrotic changes within the femoral head [22,23].

While our study provides valuable insights into the association between COVID-19 and avascular necrosis, several limitations must be acknowledged. The retrospective nature of the study and reliance on existing medical records may introduce inherent biases and limit the generalizability of the findings. Additionally, the relatively small sample size and single-center design may restrict the robustness and external validity of the results. Furthermore, unmeasured confounders and residual confounding variables may have influenced the observed associations.

Future research efforts should aim to address these limitations and further elucidate the mechanistic underpinnings of COVID-19-associated avascular necrosis. Prospective cohort studies with larger sample sizes and multi-center collaborations are warranted to validate our findings and explore potential risk factors and predictive biomarkers for avascular necrosis in COVID-19 patients. Longitudinal follow-up studies are also needed to assess the natural history and progression of avascular necrosis in this population and evaluate the efficacy of preventive and therapeutic interventions.

## 5. Conclusions

In summary, our study illuminates the intricate interplay between COVID-19, steroid therapy, and avascular necrosis of the femoral head. While COVID-19 infection may indeed exacerbate underlying risk factors for avascular necrosis, particularly in cases of severe illness, our findings suggest that the primary instigator of this complication lies in the effects induced by steroid therapy.

These results emphasize the crucial need for careful steroid use and close monitoring of COVID-19 patients, especially those at higher risk of avascular necrosis. Clinicians must navigate the delicate balance between the benefits of steroid therapy in reducing hyper- inflammatory responses and the risks of complications like avascular necrosis. Therefore, personalized treatment strategies and thorough evaluation are essential to enhance patient outcomes and reduce complications.

Furthermore, proactive screening and early detection strategies for avascular necrosis hold paramount significance in the realm of patient management. The timely identification of this debilitating condition not only facilitates prompt intervention but also serves to mitigate long-term morbidity and preserve overall patient well-being. By implementing systematic screening protocols and fostering heightened clinical awareness, healthcare providers can strive towards achieving optimal outcomes for individuals at risk of avascular necrosis, particularly in the context of COVID-19 infection.

Looking ahead, it is evident that further research endeavors are warranted to deepen our understanding of the mechanistic pathways linking COVID-19, steroid therapy, and avascular necrosis. By unraveling the intricate interplay between these factors, we can gain invaluable insights into the underlying pathophysiology of avascular necrosis in the context of COVID-19, thereby paving the way for the development of novel therapeutic strategies and interventions.

Moreover, the identification of predictive biomarkers holds promise in facilitating early detection and risk stratification for avascular necrosis in COVID-19 patients. Through the identification of biomolecular signatures indicative of disease susceptibility and progression, clinicians can preemptively tailor treatment regimens and implement targeted interventions, ultimately optimizing patient outcomes and reducing the burden of morbidity associated with this condition.

## Figures and Tables

**Table 1:** Patient demographics and clinical characteristics.

Patient ID	Age (years)	Sex	Comorbidities	COVID-19 Status	Steroid Treatment	Pulmonary Involvement	Bilateral Necrosis
1	45	M	Hypertension, Diabetes	Positive	Yes	Moderate	No
2	60	F	Obesity, Asthma	Negative	No	N/A	No
3	55	M	Rheumatoid Arthritis	Positive	Yes	Severe	Yes
...	...	...	...	...	...	...	...

**Table 2:** Radiographic findings and severity of avascular necrosis.

Patient ID	X-ray Findings	MRI Findings	Avascular Necrosis Severity (Ficat Classification)
1	Subchondral lucency	Crescent sign, joint space narrowing	Stage II
2	Normal	Absence of signal on T1-weighted images	Stage III
3	Sclerosis, flattening of femoral head	Patchy areas of low signal intensity	Stage IV
…	…	…	…

**Table 3:** Comparison of clinical characteristics between COVID-19-positive and negative patients.

Clinical Characteristic	COVID-19 Positive Patients (n = 26)	COVID-19 Negative Patients (n = 6)	*p*-Value
Mean Age (years)	52.3 ± 8.9	55.7 ± 7.2	0.321
Male Sex (%)	65.4	50.0	0.442
Comorbidities (%)	84.6	66.7	0.209
Steroid Treatment (%)	73.1	16.7	0.012 *
Pulmonary Involvement (%)	69.2	N/A	N/A
Bilateral Necrosis (%)	34.6	16.7	0.287

* Statistically significant at *p* < 0.05.

## Data Availability

No new data were created or analyzed in this study. Data sharing is not applicable to this article.
